# Neoadjuvant Palbociclib Treatment Impacts Cell Cycle Progression of DCIS

**DOI:** 10.3390/cancers18162630

**Published:** 2026-08-14

**Authors:** Rohith Battina, Candace Mainor, Marcel O. Schmidt, Gregory T. Gallanis, Ghada M. Sharif, Elaine Walsh, Kristen Whitaker, Ian Greenwalt, Lucy De La Cruz, Nicole Swanson, Kerrie B. Bouker, Anton Wellstein, Claudine Isaacs, Paula R. Pohlmann, Anna T. Riegel

**Affiliations:** 1 Lombardi Comprehensive Cancer Center, Georgetown University Medical Center, Washington, DC 20007, USA; rb1717@georgetown.edu (R.B.); mos6@georgetown.edu (M.O.S.); gtg12@georgetown.edu (G.T.G.); gms58@georgetown.edu (G.M.S.); ns1209@georgetown.edu (N.S.); briggsk@georgetown.edu (K.B.B.); wellstea@georgetown.edu (A.W.); isaacsc@georgetown.edu (C.I.); 2 Lombardi Comprehensive Cancer Center, MedStar Georgetown University Hospital, Washington, DC 20006, USA; candace.mainor@gunet.georgetown.edu (C.M.); elaine.m.walsh@gunet.georgetown.edu (E.W.); lucy.delacruz@medstar.net (L.D.L.C.); 3 MedStar Georgetown Cancer Institute, Washington Hospital Center, Washington, DC 20057, USA; kristen.d.whitaker@medstar.net; 4 Breast Surgical Oncology, Community Health Haymarket, University of Virginia, Charlottesville, VA 22903, USA; greenwalti@gmail.com; 5 Department of Breast Medical Oncology, University of Texas MD Anderson Cancer Center, Houston, TX 77030, USA; prpohlmann@mdanderson.org

**Keywords:** Ductal carcinoma in situ, palbociclib, CDK4/6 inhibitors, RNA-seq analysis, cellular deconvolution analysis, cell cycle progression, neoadjuvant therapy

## Abstract

**Background.** Ductal carcinoma in situ (DCIS) is a non-invasive disease of the breast. This study assessed the impact of the CDK4/6 inhibitor palbociclib on halting cell cycle progression in DCIS between diagnostic biopsy and surgical resection of the lesion. **Methods.** A dual-arm study was conducted involving 17 participants with DCIS who were given either 100 mg of palbociclib PO daily for twelve days or no treatment before surgery. FFPE tissues from the diagnostic biopsy and from surgical specimens were analyzed to assess changes due to treatment. **Results.** Palbociclib treatment significantly reduced Ki67 staining in surgical tissues. RNA-seq analysis comparing the diagnostic biopsy and surgical specimens showed significant downregulation of cellular proliferation pathways after treatment. A cellular deconvolution analysis demonstrated a non-significant trend of a treatment-induced decrease in the abundance of mature luminal epithelial cells. **Conclusions.** Short-term palbociclib monotherapy before surgical resection was safe and well tolerated in participants. Tissue analysis showed a significant reduction in proliferation markers and the downregulation of cell cycle progression genes within DCIS. These results support further investigation of palbociclib in patients with DCIS. **Trial registration**. ClinicalTrials.gov NCT03535506.

## Simple Summary

Ductal carcinoma in situ (DCIS) is a non-invasive lesion of the breast with excellent long-term outcomes for patients. However, in comparison to lumpectomy, neither the use of radiotherapy nor mastectomy has been shown to meaningfully affect breast cancer-specific mortality at 10 years, which indicates a gap in knowledge surrounding the factors leading to invasive recurrence. Given that palbociclib treatment leads to preclinical inhibition of tumor progression in vivo, a dual-arm study was conducted involving 17 participants with DCIS who were given either 100 mg of palbociclib PO daily for twelve days or no treatment before surgery. This pilot study was performed to identify if short-term palbociclib monotherapy before surgical resection was safe and well-tolerated in participants and could lead to anti-proliferative changes in DCIS, which could alter treatment strategies.

## 1. Introduction

Ductal carcinoma in situ (DCIS) of the breast is a non-invasive neoplastic disease of the luminal breast epithelium without invasion into the basement membrane [1]. In the United States, there are over 50,000 new cases of DCIS estimated each year, which represents 20% of all detected breast carcinomas [2]. Earlier studies have indicated that anywhere from 14 to 53% of DCIS may progress to invasive cancer if not treated adequately [3]. The central challenges in managing DCIS are to identify which patients require treatment, to prevent progression to invasive breast cancer, and to refine therapeutic approaches for those who benefit from intervention. However, no formal update to the diagnostic DCIS grading criteria has occurred since the 1997 Consensus Conference, which stratified DCIS by nuclear grade (low, intermediate, high) and subsequent efforts have not substantively advanced this classification system [4,5,6].

Currently, the standard of care for DCIS is breast-conserving surgery when feasible. Patients who undergo lumpectomy may be offered post-surgical radiation and may receive risk reduction endocrine therapy for up to 5 years [7,8,9,10,11]. For patients with more extensive DCIS, a mastectomy is recommended, and risk-reducing therapy may be offered for the contralateral side. However, neither the use of radiotherapy nor mastectomy has been shown to meaningfully affect breast cancer-specific mortality at 10 years, while long-term outcomes remain poor for a small subset of patients, with a 20-year breast cancer-specific mortality of 3.3% reported in a large SEER cohort of 108,196 women with DCIS [12].

Antihormonal therapies such as tamoxifen and aromatase inhibitors are approved for the treatment of patients with DCIS to reduce the risk of DCIS or invasive carcinoma recurrence following lumpectomy [13,14,15]. Furthermore, in one population study, tamoxifen was found to improve overall survival in hormone receptor (HR)-positive DCIS [16]. However, tamoxifen and aromatase inhibitors are associated with clinically significant adverse effects, including vasomotor symptoms, which may have a significant impact on quality of life. Therefore, in addition to refining diagnostic classification, there is also a need to improve therapeutic approaches for DCIS.

CDK4 and CDK6 kinases initiate the transition from the G1 to the S phase by forming a complex with cyclin D1 to phosphorylate the retinoblastoma (Rb) protein. Once phosphorylated, Rb protein releases E2F allowing it to bind to DNA and initiate transcription of cell cycle genes to enable cellular proliferation. CDK4/6 small-molecule kinase inhibitors, such as palbociclib, block cell cycle progression at the G1-S checkpoint and thus can inhibit proliferation. However, therapeutic inhibition of CDK4/6 relies on functional Rb signaling within the cell, as loss of physiological Rb repression would lead to continuous unregulated E2F activation. Therefore, all participants in the study were assessed for RB^+^ status before enrollment.

Palbociclib is a first-in-class drug that was approved as first-line treatment for metastatic HR-positive HER2-negative breast cancer at a dose of 125 mg PO daily for 21 days followed by 7 days of rest when used in combination with endocrine therapy [17,18,19]. Palbociclib was found to be well tolerated in combination with letrozole, with a median duration of treatment over 12 months [18]. The most common adverse effects at the dose of 125 mg were neutropenia, leukopenia, anemia, fatigue, and nausea [18]. In an initial phase I single-agent dose escalation study, it was found that the most severe adverse effect (AE) reported was grade 2 neutropenia following administration of 100 mg of palbociclib PO daily for three weeks, whereas grade 3 neutropenia was reported following administration of 125 mg palbociclib for a similar timeframe. In order to reduce the adverse effects from short-term palbociclib treatment, 100 mg PO daily was identified as the optimal dosage for this study [20].

In vitro studies have shown that palbociclib is highly effective at causing cell cycle arrest in the G1 phase with increased sensitivity in estrogen receptor-positive breast cancer cell lines [21]. Furthermore, preclinical palbociclib treatment of mice with xenograft tumors grown from human MCFDCIS cells showed inhibition of DCIS progression and reduced proliferation [22]. Therefore, we sought to determine if palbociclib treatment could inhibit further DCIS growth in the pre-operative setting.

For this trial, participants received 100 mg palbociclib PO daily, without adjuvant endocrine therapy, for 12 days with cessation of treatment before surgical resection of the DCIS lesion. The reduced dose and duration of treatment were chosen to mitigate the known adverse effects of palbociclib and to decrease any potential impact on surgical timing. To our knowledge, the effects of palbociclib treatment in patients with DCIS have not been reported. This study was designed to understand and describe single-agent activity of palbociclib in pre-invasive lesions such as DCIS and to provide critical preliminary data supportive of future studies. We sought to determine whether systemic clinical treatment with palbociclib would reveal a discernible effect on the DCIS tissue in patients. The planned readouts of the trial were based on analysis of the tissues harvested at different time points throughout the trial.

## 2. Materials and Methods

### 2.1. Sex as a Biological Variable

Both male and female participants were eligible for participation in the study protocol, but all participants enrolled and included in the analysis were female, presumably due to the greater prevalence of DCIS in women.

### 2.2. Clinical Trials

All participants enrolled in this study signed informed consent forms prior to any assessments and procedures being conducted, and all patients who met the inclusion criteria were enrolled. The participants had to be ≥18 years old with a current pathological diagnosis of DCIS of the breast of any receptor status with positive RB status by IHC in the DCIS component of the lesion. An FFPE tumor tissue block from the participants’ diagnostic biopsy had to be transmitted to the MedStar Georgetown University Hospital Pathology Department repository prior to enrollment. The participants had to be willing to receive definitive surgical therapy for DCIS and willing to provide a sample of tissue collected at definitive surgery for research. ECOG performance status had to be (0–1). Surgery was performed on study days 14–16. For participants in Group A, surgery occurred 2–4 days following cessation of palbociclib treatment. A complete blood count, comprehensive metabolic panel, and additional blood sample were taken at pre-treatment, pre-surgery, and post-surgery time points for Group A and taken at pre-surgery and post-surgery time points for Group B. The trial was open at two clinical sites: Medstar Georgetown University Hospital and MedStar Washington Hospital Center, where the samples were collected. Patients were recruited/referred to the clinical trial by breast surgeons at MedStar clinics. The palbociclib treatment was dispensed to the patient by the research pharmacy. The medication was self-administered at home, and patients were instructed to bring their completed pill diaries to the pre-operative visit for accountability purposes as they were being used to track compliance throughout the study.

For palbociclib treatment, participants had to have:

• Absolute neutrophil count ≥ 1500/mm^3^
• Platelets ≥ 120,000/mm^3^
• Hemoglobin ≥ 10 g/dL
• Total serum bilirubin ≤ ULN,
• Aspartate aminotransferase (AST or SGOT) and alanine aminotransferase (ALT or SGPT) ≤ 1.5 × institutional ULN
• Serum creatinine within normal institutional limits or creatinine clearance ≥ 50 mL/min/1.73 m^2^ for participants with serum creatinine levels above institutional ULN
• Serum or urine pregnancy test must be negative within 14 days of treatment start

Exclusion criteria for participants included concurrent therapy with other investigational products, invasive carcinoma present in the diagnostic biopsy, uncontrolled intercurrent illness, therapy with any CDK inhibitor within the past 3 months, and hormone therapies within 4 weeks of the diagnostic biopsy. Group assignment for this study was not randomized.

To be excluded from palbociclib treatment, participants must have had one or more of the following:

• A history of allergic reaction to compounds similar to palbociclib
• A condition that would interfere with absorption of palbociclib
• Current use of combination antiretroviral therapy
• Current receipt of medications that are potent inhibitors or inducers of CYP3A isoenzymes within 7 days of enrollment or during participation
• A clinically significant history of liver disease

Palbociclib treatment was to be terminated for any of the following reasons:

• Participant withdrew consent
• Unacceptable toxicity or unacceptable adherence to palbociclib
• Changes in the participant’s condition that rendered the participant not a candidate for further treatment in the opinion of the treating investigator

For safety assessment, palbociclib was used at a lower dose of 100 mg PO daily for 12 consecutive days. In the event of significant treatment-related toxicity, administration of palbociclib would have been discontinued. Any grade 3 or higher toxicity would trigger treatment discontinuation. In the case of concurrent occurrence of >3 × ULN ALT and 2 × ULN total bilirubin, at any time during the trial, palbociclib would be permanently discontinued. All adverse events were recorded (see Supplementary Table S3).

Palbociclib was stored at 20–25 °C and provided without charge to the participants or MedStar Georgetown University Hospital by Pfizer. Palbociclib drug substance and drug product were manufactured, labeled and packed according to current Good Manufacturing Practice (GMP) and Good Clinical Practice (GCP) guidelines for use in the clinical studies. Each lot of palbociclib for clinical studies was subjected to a series of quality control tests to confirm its identity, purity, potency, and quality.

### 2.3. Immunohistochemistry

FFPE tissue was stored at room temperature. IHC analyses for Ki67 (Biocare, Pacheco, CA, USA, CRM325) were performed on cut 5 µm sections from formalin-fixed paraffin-embedded (FFPE) tissue using a DAKO Agilent autostainer (Agilent, Carpinteria, CA, USA). Images were captured using an Olympus BX40 microscope (Olympus Corporation, Tokyo, Japan), and quantification of Ki67 staining was performed using QuPath positive cell detection. Diagnostic biopsy samples were stained for ER, PgR, and Rb status in a CLIA-certified laboratory.

### 2.4. FFPE RNA Extraction Methodology

RNA sequencing was performed by Novogene Corporation Inc. (Sacramento, CA, USA). RNA samples were isolated from FFPE tissues from five 5 µm sections per sample, enriched for the human exome (TruSeq RNA Exome, Illumina, San Diego, CA, USA), and sequenced on a NovaSeq X Plus platform (Illumina, San Diego, CA, USA) with a 150 paired-end read length. The output was, on average, 77 million reads per sample.

### 2.5. RNA-Seq Methodology

FASTQ files were quality-filtered with a Phred score cutoff of 20 and adapter-trimmed using TrimGalore v2.0, and reads were aligned to the human genome (hg38) and quantified using the Salmon 1.10.0 tool. Differential gene expression analysis was conducted in RStudio v4.5 with the edgeR 4.6.3 package. Low-expressing genes were filtered out. The minimum individual count for each gene in one sample had to be ≥10 or the minimum total count in all samples had to be ≥15; otherwise, they were filtered out.

### 2.6. GSEA and CIBERSORTx Methodology

Gene Set Enrichment Analysis was performed using MSigDB Hallmark gene sets [23,24,25]. CIBERSORTx was used to estimate the relative abundance of mammary cell types [26]. A custom signature matrix for the mammary epithelium was generated in CIBERSORTx using single-cell RNA-seq data from publicly available Tabula Sapiens datasets [27].

### 2.7. Statistics

Quantitative analysis of Ki67 staining was performed using non-parametric Wilcoxon/Mann–Whitney tests. The RNA-seq significance threshold was set at an FDR q-value of 0.50.

### 2.8. Study Approval

Written informed consent was obtained from each participant before enrollment. The protocol was reviewed and approved by the local Institutional Review Board in 2018 under protocol number 2018-0075.

## 3. Results

### 3.1. Study Design and Baseline Characteristics of Participants

**Figure 1:**
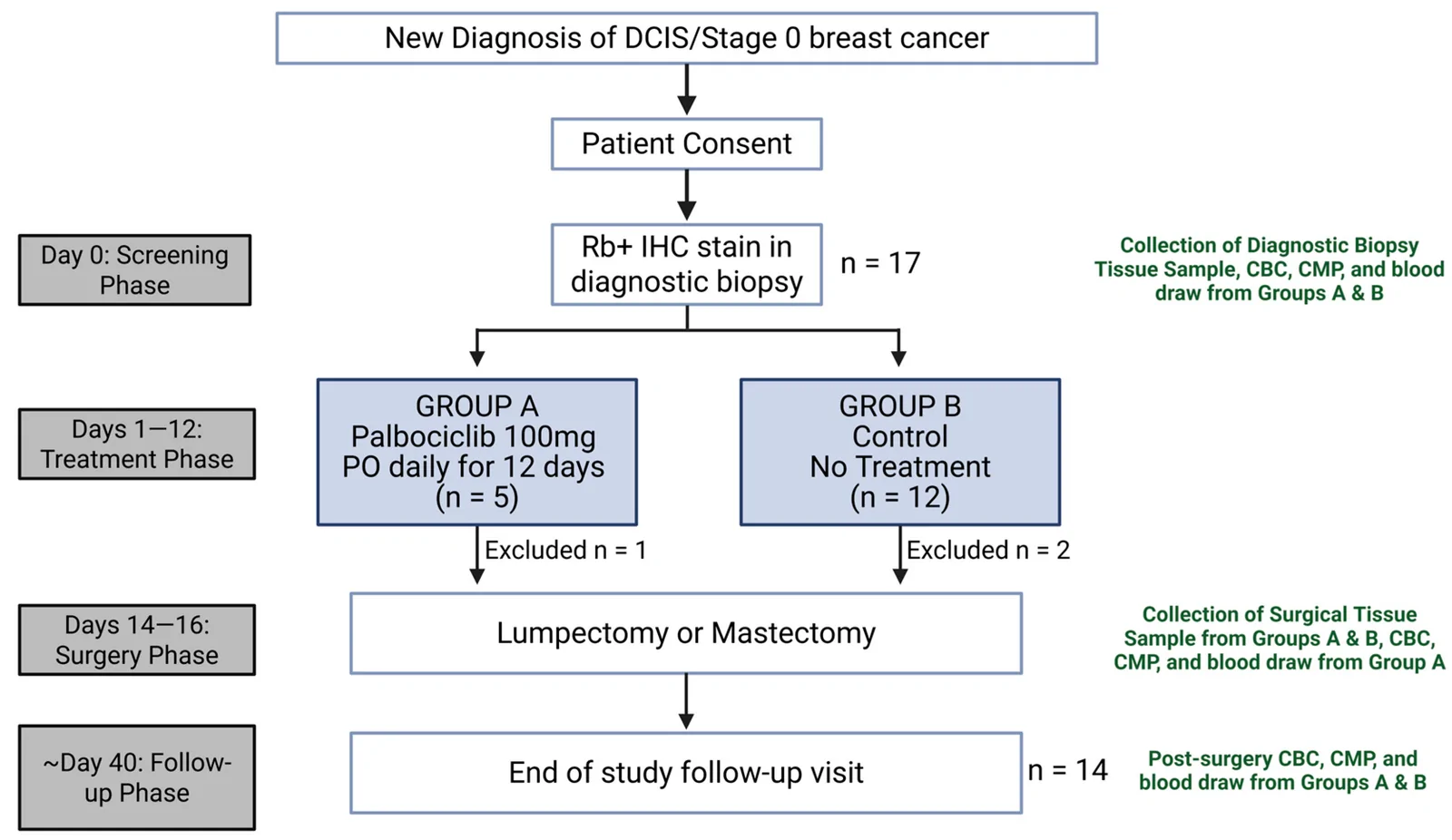
Clinical Study Timeline. Flow chart illustrates the overall timeline for participants enrolled in the clinical trial (NCT03535506). Participants were given the option to choose between Group A (Palbociclib) or Group B (Control) followed by surgery. Out of the 17 participants analyzed in the trial, 5 chose Group A (Palbociclib), and 12 chose Group B (Control). After exclusions, final biochemical analysis was performed on 10 participants in the control group and 4 participants in the treatment group. To enroll in the trial, RB^+^ stain in the biopsy sample during the screening phase was required. Additional inclusion and exclusion criteria can be found in the methods section. Created in BioRender. Battina, R. (2026) https://BioRender.com/6s41uku.

The protocol was reviewed and approved by the local Institutional Review Board in 2018 under protocol number 2018-0075. Each participant received detailed information regarding the study and signed written informed consent forms prior to undergoing any study procedures. A total of 17 participants with confirmed RB^+^ staining of DCIS biopsy tissue were enrolled in the study (NCT03535506) between 2019 and 2023 (Figure 1). Assignment to the untreated control or palbociclib treatment was based on participant choice. Twelve participants selected the control group (Group B) and did not undergo any systemic treatment prior to surgery. Five participants selected Group A and were treated with palbociclib 100 mg PO daily over the course of 12 days prior to definitive surgery.

Definitive DCIS surgery was conducted on study days 14–16, with participants undergoing either mastectomy or lumpectomy based on standard clinical criteria. In Group A, surgery occurred 2–4 days after treatment completion. The median duration between the initial dose of palbociclib and definitive surgery was 14 days. Participants were then followed during the post-operative period in the outpatient setting to ensure successful recovery. Serum and tissue samples were collected from all participants during the screening phase and the surgical phase for translational analysis. A complete blood count (CBC) was performed on all participants during the screening phase, at pre-surgery for Group A, and at the post-surgery follow-up to analyze changes due to treatment. The post-surgery follow-up visit was conducted between study days 24 and 40 and took place before the start of any post-operative radiation or endocrine therapy.

In the palbociclib cohort, one participant was included in the safety and tolerability analysis but was excluded from further analyses due to a lack of sufficient samples. At surgery, another patient who was treated with palbociclib did not show evidence of DCIS pathology in the surgical specimen. In the biopsy, foci with micropapillary DCIS and atypical ductal hyperplasia approaching DCIS were noted. However, the surgical specimen presented with focal atypical ductal hyperplasia and flat epithelial atypia, but the tissue did not show the same extent of proliferative changes in DCIS as noted in the biopsy.

In the control cohort, two participants were excluded from the analysis due to a lack of sufficient samples. There were a total of 10 participants in the control group and 4 participants in the palbociclib group who were qualified for this analysis.

Participants’ ages ranged from 30 to 79 years (Supplementary Table S1). Hormone receptor status (ER/PR) was known for all participants from the initial pathological analysis of the diagnostic biopsy (Supplementary Table S2). The majority of participants had HR-positive DCIS.

### 3.2. Safety and Tolerability

Adverse events occurred more frequently in the palbociclib treatment group, but toxicities were predominantly grade 1 and had resolved by the end of participation. All five participants completed the 12-day course of pre-operative palbociclib. There was only one instance of a reported grade 2 unrelated adverse event (Supplementary Table S3).

**Figure 2:**
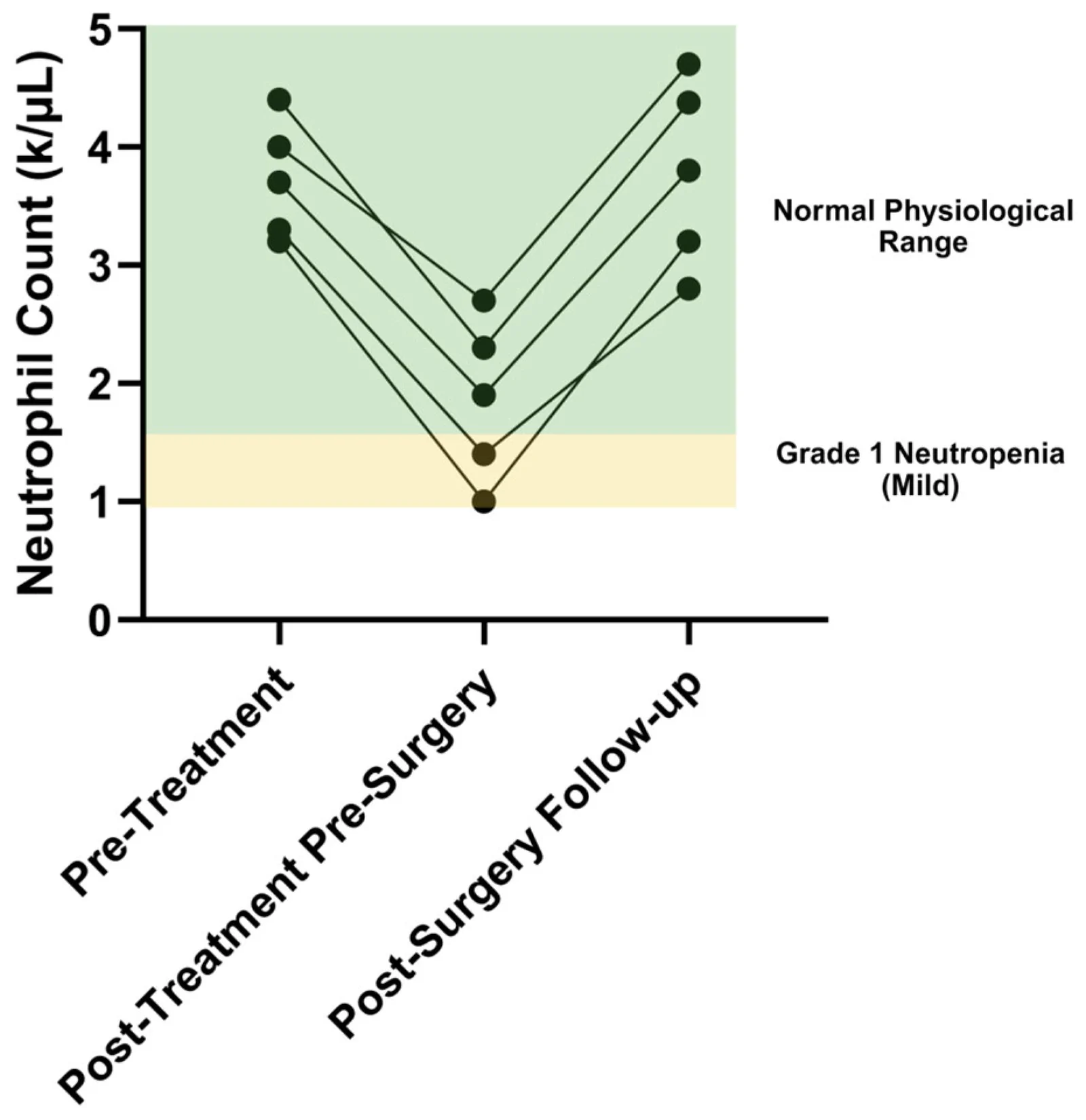
Neutrophil counts for participants in the palbociclib arm (Group A) over the course of treatment.

For safety, a CBC panel was performed for all Group A participants prior to surgery. There was a decrease in neutrophil counts in all five participants treated with palbociclib (Figure 2). Two of the five participants had absolute neutrophil levels (ANC) that fell below the normal baseline levels (1.7–8.1 k/µL) and were classified as grade 1 neutropenia, while the other three dropped but stayed within the normal physiological range. No participants experienced neutropenic fever. Mild neutropenia did not result in any surgical complications or delays. At the follow-up appointment, neutrophil counts in all participants had recovered to baseline levels. Total white blood cell count was reduced at the pre-surgery post-treatment time points in all five participants, indicating mild leukopenia; this also recovered by the follow-up appointment. One participant experienced mildly decreased hemoglobin (10.7 g/dL, normal range: 11–14.5 g/dL) and hematocrit (32.8%, normal range: 34.5–44%) and another participant had mild thrombocytopenia (133 k/µL, normal range: 145–400 k/µL). Other immune cell populations and erythrocyte markers were not significantly affected by palbociclib treatment and were within control levels following the twelve-day course of treatment.

### 3.3. Measuring Cellular Proliferation Using Ki67 Biomarker

**Figure 3:**
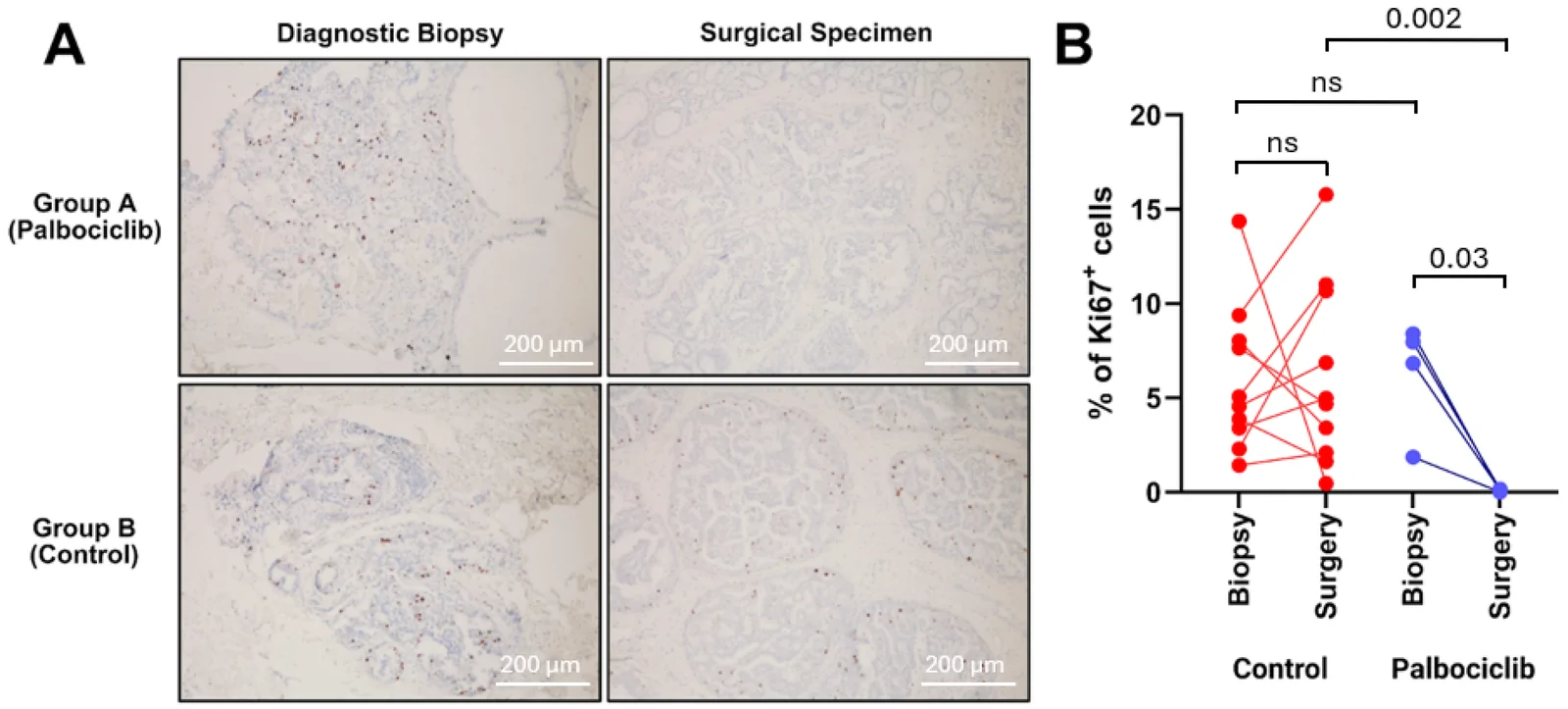
Reduction in Ki67 staining following 12 days of treatment with palbociclib. (**A**) Representative immunohistochemical (IHC) staining for the cellular proliferation marker, Ki67, pre- and post-treatment. (**B**) Quantification for % of cells positively stained for Ki67 for Control Biopsy (5.998 ± 1.233%), Control Surgery (6.156 ± 1.554%), Palbociclib Biopsy (6.257 ± 1.505%), and Palbociclib Surgery (0.0796 ± 0.0398%). Paired *t*-tests were performed for specimens from the same participant within the group: Palbociclib Biopsy vs. Surgery, *p* = 0.0252, Control Biopsy vs. Surgery, *p* = 0.9406 (ns). Mann–Whitney non-parametric tests were performed for comparisons between groups: Control Biopsy vs. Palbociclib Biopsy, *p* = 0.8392 (ns), Control Surgery vs. Palbociclib Surgery, *p* = 0.002).

FFPE tissues from the diagnostic biopsy and from definitive surgery specimens were processed and stained for Ki67, a nuclear marker of cellular proliferation (Figure 3A). In the diagnostic biopsy samples, there was no discernible difference in the percentage of cells staining for Ki67 between groups (Figure 3B). However, as expected, there was a significant decrease in Ki67-positive cell staining in participants’ samples after palbociclib treatment (*p* = 0.0252). There was also a significant decrease in Ki67 staining when comparing palbociclib and control surgical specimens (*p* = 0.002). This indicates that treatment with palbociclib significantly reduced Ki67 within DCIS tissue after 12 days of treatment.

### 3.4. RNA-Seq Analysis

To evaluate molecular changes induced by palbociclib treatment, we performed a transcriptome analysis of the FFPE tissue sections by RNA-seq. RNA was extracted from five consecutive 5 µm sections obtained from each participant’s tissue specimen at the scheduled time points.

**Figure 4:**
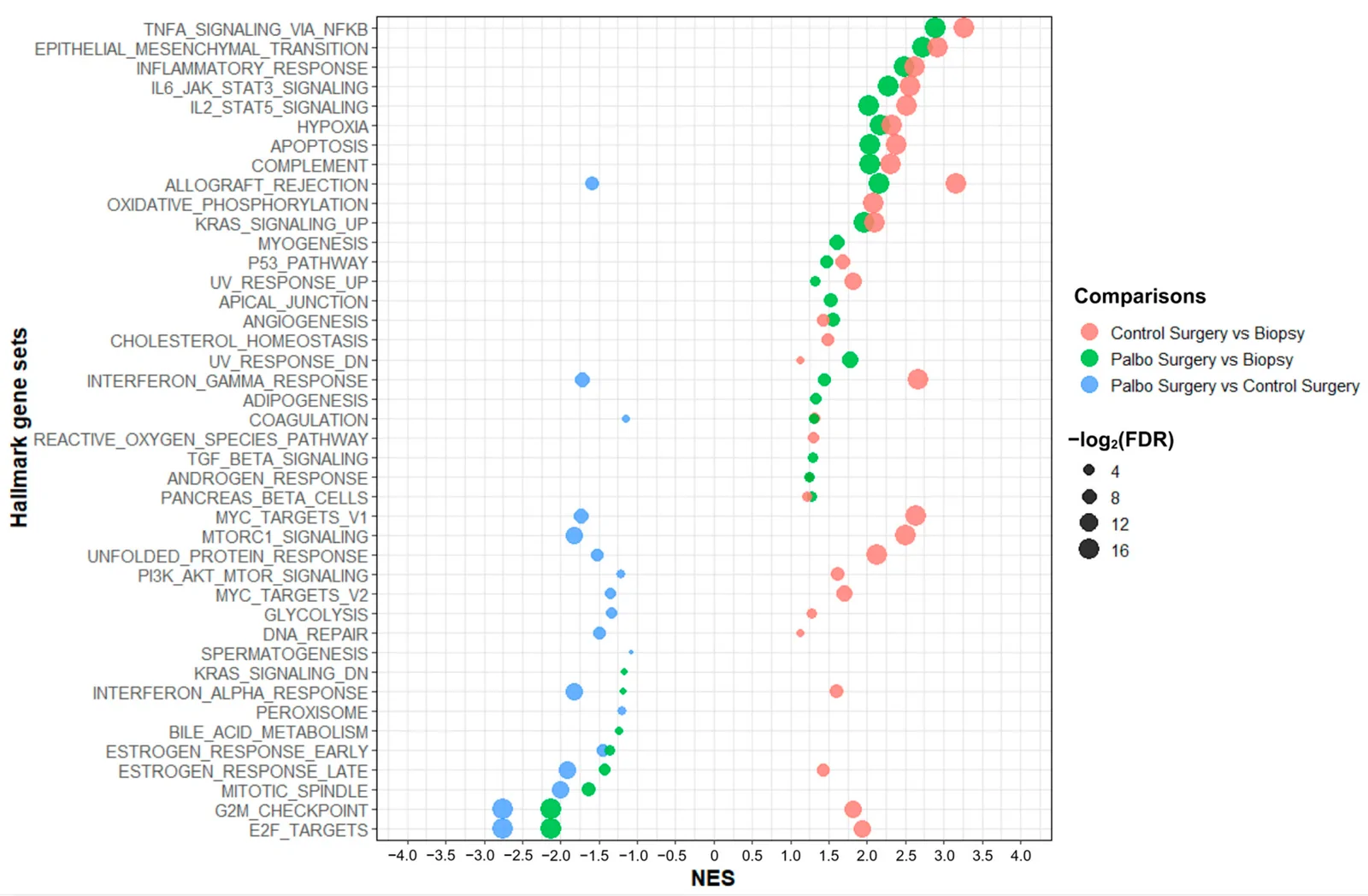
Gene Set Enrichment Analysis (GSEA) of bulk RNA-seq data from diagnostic biopsy and post-surgery FFPE tissue samples. RNA-seq analysis revealed significant downregulation of multiple cell cycle and cellular proliferation pathways in surgical specimens after 12 days of treatment with palbociclib (green). Normalized Enrichment Score (NES) and associated statistical significance for all pathways with a false discovery rate (FDR) q-value ≤ 0.5. The E2F targets, G2M checkpoint, mitotic spindle, estrogen response, IFN-α, and IFN-γ pathways had FDR q-values < 0.25 (see Supplementary Table S4 for full values). Comparisons were also made between control surgical samples versus diagnostic biopsy (red) and directly between surgical samples for palbociclib treatment and control (blue).

All initial diagnostic biopsy sample data were pooled into one group (Biopsy) for comparison against definitive surgery samples obtained after palbociclib treatment (Group A) or no systemic treatment (Group B). Gene Set Enrichment Analysis (GSEA) revealed significant downregulation of expression in multiple established Hallmark gene sets that encompass the cell cycle and cellular proliferation, such as G2M checkpoint, E2F targets, and mitotic spindle, after treatment with palbociclib (Figure 4).

**Figure 5:**
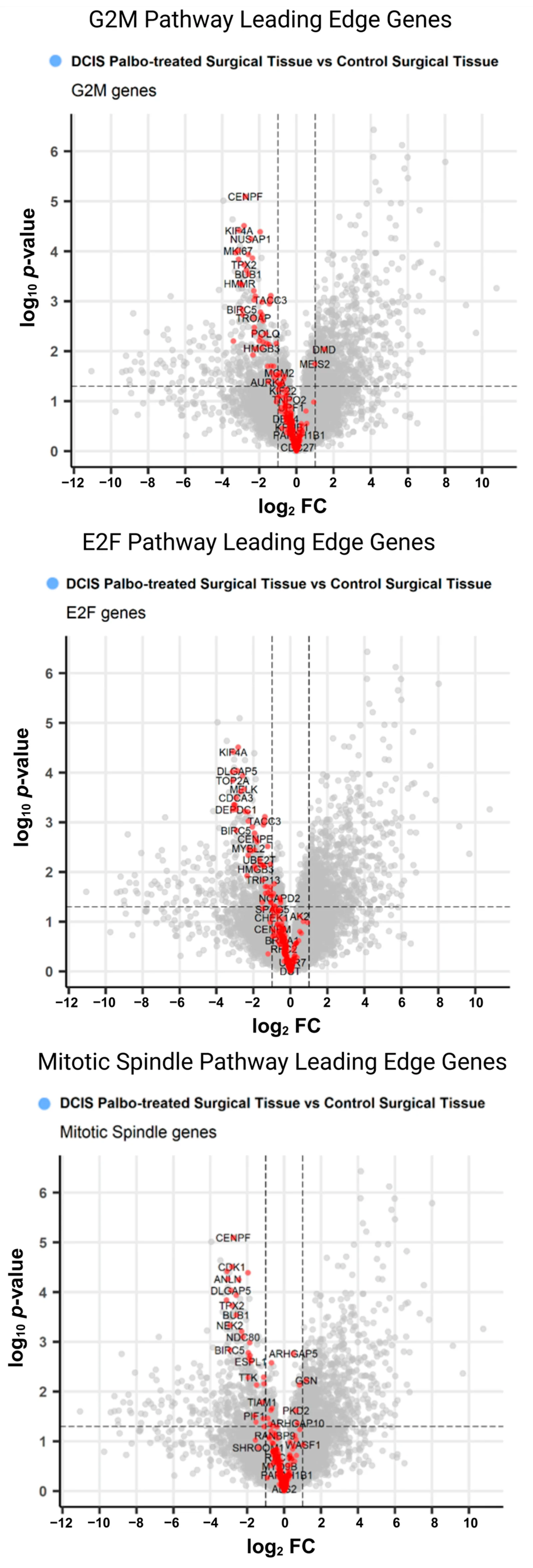
Decreased gene expression of leading-edge genes for cell proliferation signaling pathways after palbociclib treatment. Volcano plots of bulk RNA-seq data from biopsy and post-surgery FFPE tissue samples. Comparison of gene expression in G2M, E2F and mitotic spindle pathways between palbociclib treatment and control at surgery shows downregulation in the majority of leading-edge genes. Leading-edge genes are highlighted in red and labeled. Dashed lines indicate genes with fold change (FC) > 2 and *p*-value < 0.05.

Further examination of the leading-edge genes in these pathways demonstrated a sweeping downregulation of expression throughout distinct pathways in palbociclib versus control samples after treatment (Figure 5). In the G2M Hallmark gene set, there was a significant decrease in the fold change of multiple genes known to play a role in cellular division, including *CENPF*, *KIF4A*, *TPX2*, *NUSAP1*, *BUB1*, and *HMMR*. In the E2F target gene set, there was a similar downregulation of genes involved in cell cycle progression, including *KIF4A*, *TOP2A*, *DLGAP5*, *CDCA3*, *BIRC5*, and *MYBL2*. For the mitotic spindle gene set, there was a downregulation of *CENPF*, *CDK1*, *DLGAP5*, *BUB1*, *NEK2*, and *TTK* as well as many others. This is consistent with the strong inhibitory effect of palbociclib on CDK4/6. In addition, the estrogen response early and estrogen response late gene sets were downregulated. All DCIS lesions from participants enrolled in the study were found to be ER+ on IHC staining; thus, palbociclib treatment could play a dual role by downregulating baseline cell cycle signaling as well as through its effects on estrogen-induced proliferation.

Interestingly, palbociclib treatment also led to the downregulation of IFN-α and IFN-γ signaling pathways. This was surprising as CDK4/6 inhibition has been linked to anti-tumor immunity by stimulating the production of type III interferons and enhancing tumor antigen presentation [28]. Furthermore, aberrant IFN-signaling pathways have been associated with acquired resistance to palbociclib [29].

Palbociclib treatment did not significantly change gene expression in other pathways involved in tumor progression, such as the inflammatory response, apoptosis, and epithelial–mesenchymal transition.

Surprisingly, the comparison between the diagnostic biopsy and surgical specimens in the untreated control group showed an increase in the expression of leading-edge genes in the previously discussed cell cycle pathways. This indicates that further growth of the DCIS lesion could have been ongoing during the short observation period (Supplemental Figure S1). This finding is in stark contrast to the comparison between the diagnostic biopsy and surgical samples in the palbociclib-treated group, where we observed a downregulation in the majority of leading-edge genes within these pathways.

### 3.5. Cellular Deconvolution Analysis

In addition to the above pathway analysis, a cellular deconvolution analysis was performed using CIBERSORTx to identify the relative cellular abundance within the biopsy and tumor samples [26]. CIBERSORTx analyzes bulk RNA sequencing reads from a tissue sample in order to determine the relative abundance of various cell types within the original sample using an atlas of single-cell reference data. We used publicly available reference data from *Tabula sapiens* to impute gene expression profiles for cell types within the human breast.

**Figure 6:**
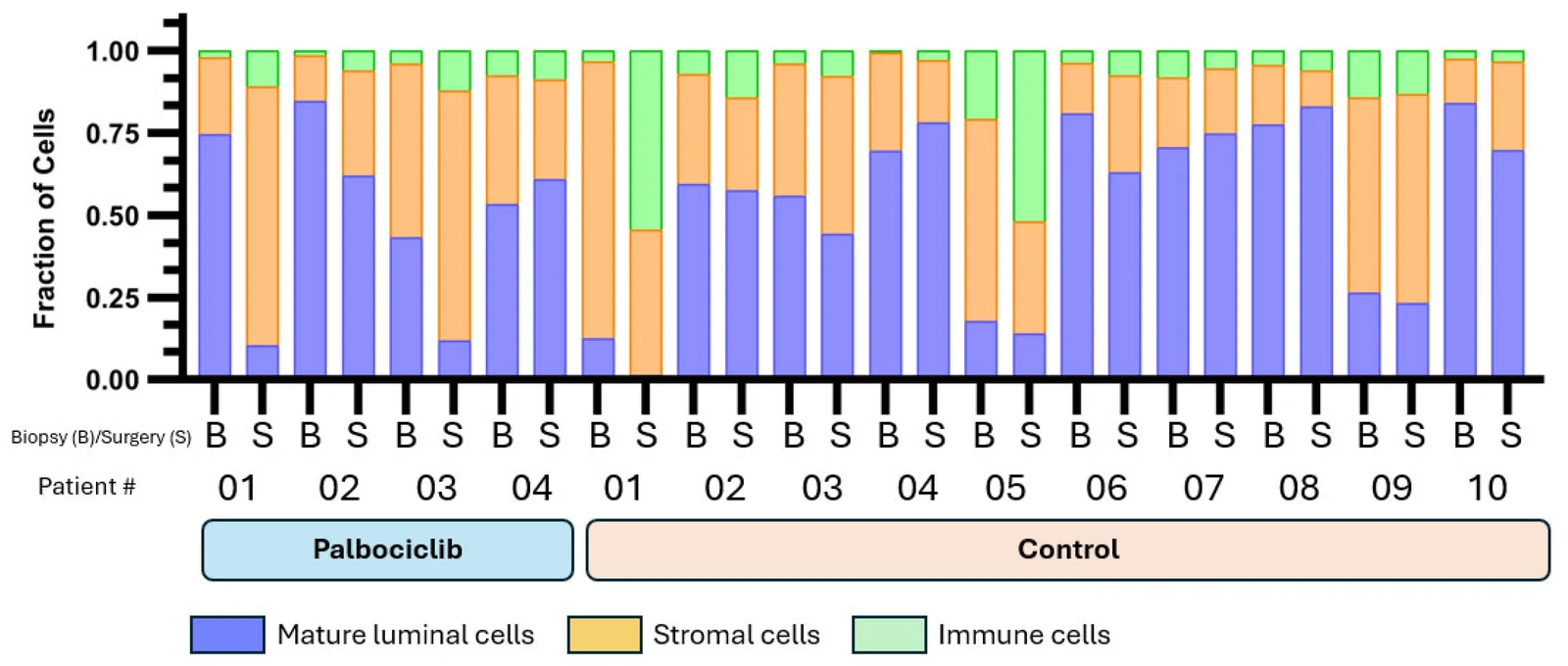
Cellular deconvolution analysis comparing biopsy (B) and surgical (S) DCIS tissue from each individual participant. CIBERSORTx analysis of bulk mRNA from the tissue specimen. Three of four participants treated with palbociclib showed a significant decrease in relative abundance of mature luminal cells between biopsy and surgery. The immune cell category was composed of the following cell types: macrophages, NK, T, B, mast, and plasma cells. The stromal cell category was composed of basal cells, venous/arterial endothelial cells, pericytes, lymphatic endothelial cells, lipofibroblasts, and luminal progenitor cells.

The comparison demonstrated a trend with three of the four participants treated with palbociclib having a decrease in the relative abundance of mature luminal cells following treatment. This is indicative of a decrease in the presence of DCIS cells that originate from the luminal epithelium (Figure 6). Notably, in the control group, the relative abundance of mature luminal cells stayed more consistent between the diagnostic biopsy and surgical tissue specimens. Due to the low sample size and inter-sample variability, there was no significant difference between groups, but this trend suggests that palbociclib treatment could have an effect on the abundance of mature luminal cells.

In summary, a twelve-day treatment with single-agent palbociclib before surgical resection was safe, well-tolerated, and biologically active in participants. Importantly, palbociclib treatment led to a significant reduction in the cellular proliferation marker Ki67 and downregulation of expression in associated cellular proliferation genes within DCIS tissue, while the untreated control group had indicators of continued growth of the DCIS lesion. This confirms that palbociclib treatment is biologically active in DCIS, even with short-term treatment prior to surgery.

## 4. Discussion

### 4.1. Study Limitations

The major limitation of this study was the sample size. While the results appeared to be significant based on IHC and RNA-seq analyses, the small sample size is a noteworthy constraint. Due to the small sample size, it is important to emphasize that this reduces the strength of the conclusions we can draw, making it more difficult to generalize these findings to the larger population with RB^+^ DCIS lesions; thus, clinical benefit requires further investigation in a larger cohort. Additionally, participants self-selected their treatment group rather than being randomized, which could also reduce the generalizability and strength of the findings.

Although in a different disease setting, enrollment in this trial was hindered by the concomitant emergence of negative findings from major trials assessing palbociclib in early-stage breast cancer. The PALLAS clinical trial was conducted to determine if adjuvant palbociclib treatment with endocrine therapy could improve invasive disease-free survival (iDFS) in patients with early-stage HR-positive HER2-negative breast cancer. There was no statistically significant difference compared to endocrine therapy alone [30]. Similarly, in the PALLET clinical trial, neoadjuvant palbociclib with endocrine therapy in estrogen receptor-positive breast cancer did not demonstrate significant differences in clinical response [31]. It should be noted that our study did not involve the use of endocrine therapy and was performed using a lower dose with shorter duration. This is commented on further in the Discussion.

Another limitation was that participants expressed concern about the increased risk of neutropenia in the setting of the COVID-19 pandemic, which could have reduced their willingness to enroll in arm A.

### 4.2. Discussion

The biological impact of twelve days of 100 mg PO palbociclib monotherapy before surgical resection of DCIS tissue was investigated. Palbociclib treatment was found to be well tolerated, with mainly transient grade 1 toxicities. Two of the five participants treated with palbociclib experienced reversible mild neutropenia that recovered following cessation of treatment. None of the participants experienced neutropenic fever or any complications that would normally result in a surgical delay. In contrast, the PALLET trial found that 49.8% of patients treated with palbociclib and letrozole for up to 14 weeks experienced grade 3 or higher toxicity. This study found that a much shorter course of palbociclib did not lead to such high-grade toxicities. Therefore, short-term palbociclib treatment will not strongly contribute to overtreatment or significant adverse effects in patients with an appropriate indication for treatment, in comparison with current long-term palbociclib use as first-line treatment for metastatic HR-positive, HER2-negative breast cancer, which has been associated with significant adverse effects.

A 12-day course of low-dose palbociclib treatment led to a decrease in proliferation markers within the DCIS lesion. Ki67 is a well-known marker for cancer that is found to be present in proliferating cells but absent in quiescent cells. The treated and untreated groups had similar levels of Ki67 staining prior to treatment, but there was a significant difference in Ki67 staining between groups in the surgical specimens, indicating that palbociclib treatment had an effect.

It is clear that broadly treating all DCIS patients with low-dose palbociclib for 2 weeks would still risk contributing to overtreatment in a disease where the 20-year breast cancer-specific mortality is 3.3% [12]. However, it is important to consider that patients with high-grade DCIS lesions have been shown to have a significantly higher risk of developing distant metastases [32]. Thus, palbociclib treatment could have clinical benefit as neoadjuvant treatment for patients with high-grade lesions by halting proliferation and reducing the risk of recurrence. However, determining whether treatment of high-grade lesions is an appropriate indicator for palbociclib therapy should be explored further in a future randomized clinical trial with a larger sample size and better stratification.

Interestingly, a previous study identified that 2 weeks of endocrine therapy also led to lower Ki67 expression in primary breast cancer and was significantly associated with a higher recurrence-free survival rate [33]. This demonstrates that even short-term treatment courses can have a clinical benefit in breast cancer.

This was corroborated by analysis of the transcriptome through Gene Set Enrichment Analysis, which showed a downregulation of genes in multiple Hallmark pathways known to regulate cell cycle progression following treatment. Furthermore, when comparing diagnostic biopsy and surgical specimens from untreated participants in the control group, there was increased expression within these cell cycle pathways. These proliferative signaling changes could be explained by ongoing DCIS progression between the two tissue collection time points and by a post-biopsy wound healing response. There was increased transcription of multiple Hallmark pathways linked to wound healing, such as the epithelial–mesenchymal transition and inflammatory response in both surgical groups in comparison to the biopsy samples. Nevertheless, there was a significant decrease in cell proliferation pathway expression with palbociclib treatment. It is important to acknowledge that no patients with high-grade DCIS identified on biopsy selected the palbociclib arm, which could have influenced the large difference in gene expression changes between the two treatment groups at surgery and affected the comparison between groups for the GSEA. However, these results mirrored preclinical findings in murine models of DCIS treated with palbociclib [22]. We also saw a significant downregulation of the estrogen signaling pathway, suggesting that the ideal cohort for further study would be patients found to have ER/PR+ DCIS lesions with intact RB signaling to target cell cycle and estrogen signaling leading to proliferation and metastasis.

Surprisingly, palbociclib treatment also led to the downregulation of the IFN-α and IFN-γ signaling pathways. A recent study reported in abstract form described similar findings in patients with early-stage breast cancer who were treated with CDK4/6i monotherapy for 14 days, where responders to treatment had suppressed IFN-α and IFN-γ pathways compared with non-responders [34]. Given the potential therapeutic implications for anti-tumor immunity, this interaction of CDK4/6 inhibition needs to be further explored.

Finally, a cellular deconvolution analysis showed that a decrease in the relative abundance of mature luminal cells indicates that palbociclib treatment targets the cellular proliferation of DCIS. Three of the four participants had a large decrease in the relative abundance of mature luminal cells. This was surprising as palbociclib is mainly known to induce cell cycle arrest and not cell death, though induction of senescence could lead to the recruitment of innate immune cells [35].

Notably, one participant had low-grade DCIS identified at biopsy, yet no residual DCIS was detected at surgery following palbociclib treatment, consistent with a marked reduction in proliferative changes. This raises the possibility that palbociclib monotherapy could lead to the elimination of DCIS in a subset of patients, but given the small sample size, this will need to be validated in a larger study. Furthermore, no patients treated with palbociclib were found to have an increase in tumor grade following treatment, whereas two patients in the control group demonstrated changes in tumor grade within the two weeks between biopsy and surgery, suggesting further progression of the lesions (Supplementary Table S2).

Despite palbociclib being regarded as a cytostatic agent in early-stage breast cancer, this observation raises the possibility that palbociclib monotherapy may eliminate DCIS in a subset of patients with low-grade DCIS, although confirmation will require evaluation in a larger cohort. It is also possible that a short course of treatment could help further stratify risk in this heterogeneous disease, informing future individualized treatment selection; however, this hypothesis will require evaluation in a larger trial.

## 5. Conclusions

It is important to highlight that this trial was intended to be a short pharmacodynamic study with a small cohort to determine if further investigation of palbociclib should be considered in DCIS. We did not identify any discernible toxicity from the short duration of treatment and found some interesting results, as highlighted above. Based on these results, it is clear that further study of palbociclib treatment in a larger cohort is needed to determine the clinical relevance of these findings and identify whether monotherapy can lead to the elimination of DCIS and reduce long-term recurrence, given the small sample size in this preliminary study. Currently, endocrine therapy is the only approved therapy for DCIS; if short-term palbociclib treatment could lead to the elimination of DCIS in an appropriately selected subset of patients, it could reduce the need for increased surgical treatment followed by post-surgical therapy.

## Data Availability

The original RNA-sequencing data presented in this study was uploaded to GEO Accession Number: GSE324721 with patient consent.

## Supplementary Materials

The following supporting information can be downloaded at https://www.mdpi.com/article/10.3390/cancers18162630/s1, Figure S1: Volcano plot comparison of Group B surgical tissue vs. biopsy and Group A surgical tissue vs. biopsy; Table S1: Age/Demographics for participant cohort in clinical trial NCT03535506; Table S2: Hormone Receptor Status and Nuclear Grade of Participant Tumors determined by IHC during pathological examination of diagnostic biopsy pre-enrollment; Table S3: Clinical Trial Adverse Events Table. Table S4. GSEA Hallmark Pathways with NES and FDR q-values.

## Abbreviations

The following abbreviations are used in this manuscript:

DCIS	Ductal Carcinoma in Situ
FFPE	Formalin-fixed paraffin embedded
CDK	Cyclin-dependent Kinase
AE	Adverse Events
IHC	Immunohistochemistry
GSEA	Gene Set Enrichment Analysis
CBC	Complete Blood Count
