# Supplementary material for: Neoadjuvant Palbociclib Treatment Impacts Cell Cycle Progression of DCIS
Source: Cancers (Basel). 2026 Aug 14;18(16):2630. doi: 10.3390/cancers18162630 (PMC13511700; doi:10.3390/cancers18162630)
Supplement: Supplementary file 1 [file cancers-18-02630-s001.zip › cancers-4477270-supplementary.pdf]

Supplementary Table S1. Age/Demographics for participant cohort in clinical trial NCT03535506.

		Total	Group A	Group B
**Age**	30-39	3	2	1
	40-49	2	0	2
	50-59	4	1	3
	60-69	3	1	2
	70-79	2	0	2
**Gender**	Female	14	4	10
	Male	0	0	0
**Race**	American Indian or Alaska Native	0	0	0
	Asian	1	0	1
	Black or African American	6	2	4
	Native Hawaiian or Other Pacific Islander	0	0	0
	White	6	2	4
	Not Reported/Unknown	1	0	1

Supplementary Table S2. Hormone Receptor Status and Nuclear Grade of Participant Tumors determined by IHC during pathological examination of diagnostic biopsy pre-enrollment. RB status was determined by IHC of archived biopsy tissue during the screening phase. For enrollment, tumors had to stain positively for RB. All participants' tumors stained for ER and most also stained for PR.

**Palbo Arm**	ER Staining	PR Staining	RB Staining	Nuclear grade at biopsy	Nuclear grade at surgery
A-01	Yes (70%)	Yes (70%)	Yes (>90%)	Low	No DCIS noted
A-02	Yes (>90%)	Yes (27%)	Yes	Intermediate	Intermediate
A-03	Yes (>90%)	Yes (75%)	Yes (20%)	Intermediate	Intermediate
A-04	Yes (>95%)	Yes (3-5%)	Yes (80%)	Not graded	Intermediate
**Control Arm**	ER Staining	PR Staining	RB Staining	Nuclear grade at biopsy	Nuclear grade at surgery
B-01	Yes (>99%)	No (0%)	Yes (>90%)	Intermediate	Intermediate
B-02	Yes (100%)	Yes (40%)	Yes (70%)	High	High
B-03	Yes (90%)	Yes (40%)	Yes (>90%)	High	High
B-04	Yes (>95%)	Yes (35%)	Yes (20%)	Intermediate	Intermediate
B-05	Yes (90%)	Yes (90%)	Yes (80%)	Low	Low

B-06	Yes (100%)	Yes (57%)	Yes (80%)	Intermediate	Intermediate
B-07	Yes (>99%)	Yes (>90%)	Yes (>90%)	Intermediate	Intermediate
B-08	Yes (95%)	Yes (80%)	Yes (90%)	Intermediate	Intermediate
B-09	Yes (>90%)	Yes (>90%)	Yes (70%)	Intermediate	High
B-10	Yes (95%)	Yes (90%)	Yes (80%)	Low	Intermediate

Supplementary Table S3. Clinical Trial Adverse Events Table.

Participant	Arm	Causality	CTC Term	AE Description	Toxicity Grade	Outcome
A-01	Palbociclib	Probable	Dry mouth		1	Recovered
A-01	Palbociclib	Probable	Nausea	Nausea	1	Not Recovered/ Not resolved
A-03	Palbociclib	Possible	Headache	Intermittent. Relieved with OTC medication.	1	Recovered
A-03	Palbociclib	Unrelated	Headache	Continuous. Felt to be related to COVID-19.	2	Recovered
A-03	Palbociclib	Unrelated	Infections and infestations - Other, specify	Tested positive for COVID-19.	1	Recovered
A-04	Palbociclib	Possible	Gastrointestinal disorders - Other, specify	Dry Lips	1	Recovered
B-08	Control	Unrelated	Psychiatric disorders - Other, specify	Hallucinations	1	Recovered
B-10	Control	Unrelated	Breast pain	Mild soreness	1	Recovered

Supplementary Table S4. GSEA Hallmark Pathways with NES and FDR q-values.

	**Control Surgery vs Biopsy**		**Palbo Surgery vs Biopsy**		**Palbo Surgery vs Control Surgery**	
**Pathway**	**NES**	**FDR q-value**	**NES**	**FDR q-value**	**NES**	**FDR q-value**
TNFA_SIGNALING_VIA_NFKB	3.2746456	0	2.8993306	0	N/A	N/A
EPITHELIAL_MESENCHYMAL_TRANSITION	2.9167488	0	2.7255542	0	N/A	N/A
INFLAMMATORY_RESPONSE	2.6177528	0	2.4892542	0	N/A	N/A
IL6_JAK_STAT3_SIGNALING	2.5604868	0	2.2821355	0	N/A	N/A
IL2_STAT5_SIGNALING	2.5118532	0	2.028749	0	N/A	N/A
HYPOXIA	2.3270347	0	2.1735272	0	N/A	N/A
APOPTOSIS	2.3895526	0	2.0330174	0	N/A	N/A
COMPLEMENT	2.3068128	0	2.0432012	0	N/A	N/A
ALLOGRAFT_REJECTION	3.164148	0	2.1603422	0	-1.591673	0.0118573615
OXIDATIVE_PHOSPHORYLATION	2.0797167	0	N/A	N/A	N/A	N/A

KRAS_SIGNALING_UP	2.0920484	0	1.9658389	0	N/A	N/A
MYOGENESIS	N/A	N/A	1.6015625	0.0069099423	N/A	N/A
P53_PATHWAY	1.6766922	0.007255327	1.4631977	0.025534818	N/A	N/A
UV_RESPONSE_UP	1.8209629	0.00087098766	1.3145487	0.07982063	N/A	N/A
APICAL_JUNCTION	N/A	N/A	1.5226786	0.014351662	N/A	N/A
ANGIOGENESIS	1.4268776	0.029598765	1.5638661	0.010904261	N/A	N/A
CHOLESTEROL_HOMEOSTASIS	1.4781916	0.022837859	N/A	N/A	N/A	N/A
UV_RESPONSE_DN	1.1284035	0.23107338	1.7857188	0.0013651225	N/A	N/A
**INTERFERON_GAMMA_RESPONSE**	**2.6616387**	**0**	**1.4373797**	**0.029374732**	**-1.7183939**	**0.0039980114**
ADIPOGENESIS	N/A	N/A	1.335875	0.06831156		
COAGULATION	1.3192842	0.067275316	1.3024108	0.08558486	-1.1544771	0.23539622
REACTIVE_OXYGEN_SPECIES_PATHWAY	1.3019234	0.07465481	N/A	N/A	N/A	N/A
TGF_BETA_SIGNALING	N/A	N/A	1.2852596	0.094153725	N/A	N/A
ANDROGEN_RESPONSE	1.2481395	0.10054207	1.2528014	0.11250587	N/A	N/A
PANCREAS_BETA_CELLS	1.213257	0.1260164	1.275312	0.09711013	N/A	N/A
MYC_TARGETS_V1	2.6333268	0	N/A	N/A	-1.7363015	0.0032542208
MTORC1_SIGNALING	2.5002446	0	N/A	N/A	-1.8178723	0.00040909092
UNFOLDED_PROTEIN_RESPONSE	2.1217847	0	N/A	N/A	-1.5292659	0.020323148
PI3K_AKT_MTOR_SIGNALING	1.6148257	0.010928822	N/A	N/A	-1.2095959	0.1749358
MYC_TARGETS_V2	1.7132871	0.002493775	N/A	N/A	-1.3423781	0.06981265
GLYCOLYSIS	1.2768183	0.09039505	N/A	N/A	-1.3335667	0.06981521
DNA_REPAIR	1.128518	0.23852734	N/A	N/A	-1.489595	0.025275446
SPERMATOGENESIS	N/A	N/A	N/A	N/A	-1.068344	0.432215
KRAS_SIGNALING_DN	N/A	N/A	-1.1615219	0.31908423	N/A	N/A
**INTERFERON_ALPHA_RESPONSE**	**1.6006277**	**0.010432057**	**-1.1842643**	**0.30828583**	**-1.8221474**	**0.00049090915**
PEROXISOME	N/A	N/A	N/A	N/A	-1.1962842	0.17938486
BILE_ACID_METABOLISM	N/A	N/A	-1.2397448	0.22718619	N/A	N/A
**ESTROGEN_RESPONSE_EARLY**	**N/A**	**N/A**	**-1.366351**	**0.08329572**	**-1.4531871**	**0.031136123**
**ESTROGEN_RESPONSE_LATE**	**1.4277676**	**0.030832049**	**-1.4224776**	**0.060938787**	**-1.9085424**	**0.0006136364**
**MITOTIC_SPINDLE**	**N/A**	**N/A**	**-1.6328914**	**0.009072402**	**-1.997996**	**0.00081818184**

G2M_CHECKPOINT	1.8198361	0.00082259945	-2.1200337	0	-2.7584283	0
E2F_TARGETS	1.9383837	0.00092542445	-2.1274328	0	-2.7533793	0

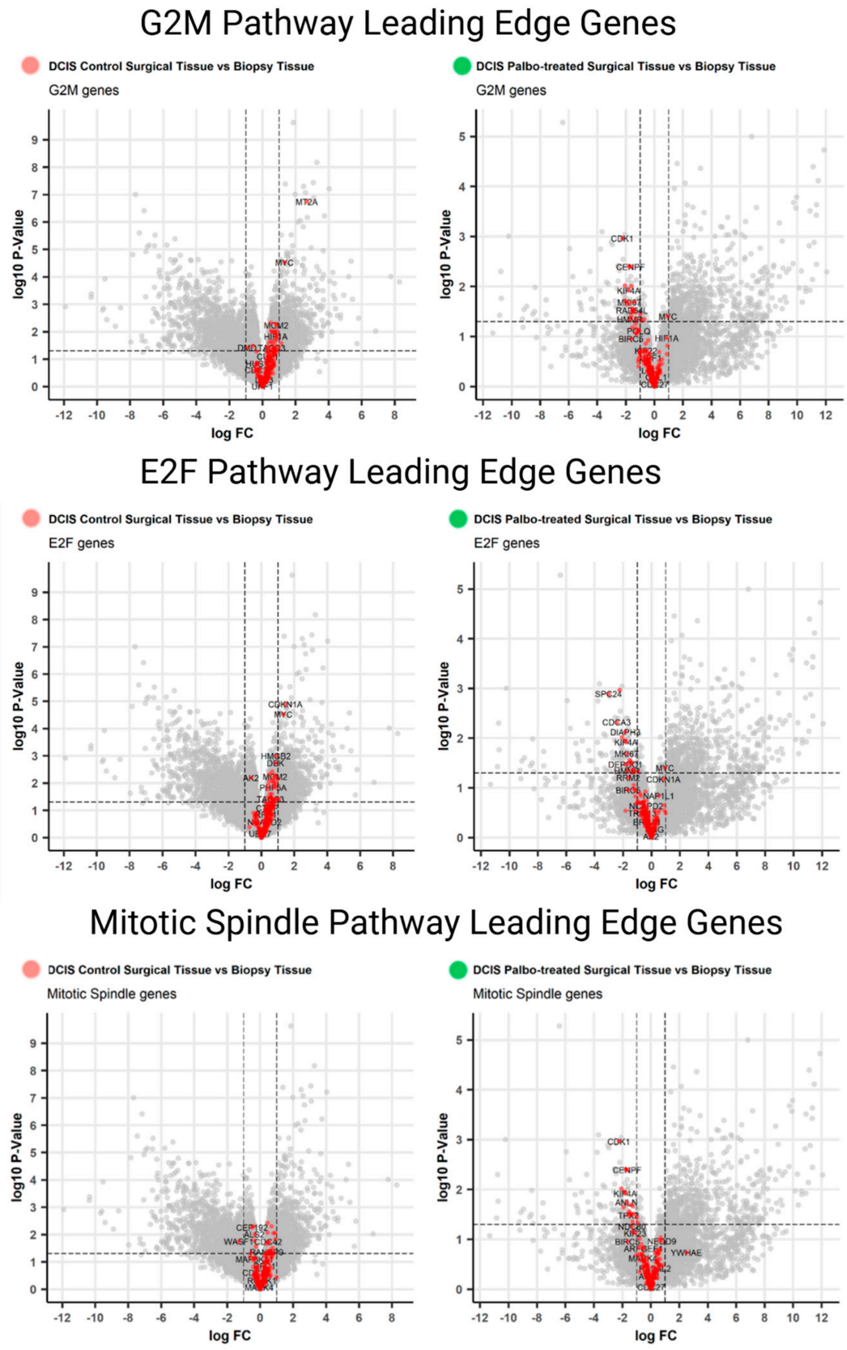

Supplementary Figure S1. Volcano plot comparison of Group B surgical tissue vs. biopsy and Group A surgical tissue vs. biopsy.
